# Predicting Prognosis of Hepatocellular Carcinoma Patients Based on the Expression Signatures of Mitophagy Genes

**DOI:** 10.1155/2022/4835826

**Published:** 2022-09-16

**Authors:** Yan-ke Li, Li-rong Yan, Li-yue Jiang, Qian Xu, Ben-gang Wang

**Affiliations:** ^1^Institute of General Surgery, The First Affiliated Hospital of China Medical University, Shenyang 110001, China; ^2^Department of Anorectal Surgery, Institute of General Surgery, The First Hospital of China Medical University, Shenyang 110001, China; ^3^Tangdu Hospital of the Fourth Military Medical University, Xi'an 711032, China; ^4^Department of Hepatobiliary Surgery, Institute of General Surgery, The First Hospital of China Medical University, Shenyang 110001, China

## Abstract

**Background:**

The unbalance of mitophagy was closely related to hepatocellular carcinoma (HCC) progression. At present, it has not been uncovered about the influence of mitophagy genes on HCC prognosis and their potential pathogenesis.

**Materials and Methods:**

The expression and clinical information of HCC in TCGA cohort were used to identify mitophagy differentially expressed genes (MDEGs) with prognostic value. The prognostic model of mitophagy genes was built and externally validated by LASSO regression in TCGA cohort and ICGC cohort, respectively. The function of the prognostic signature and its association with immune cell infiltration were explored. The profile of MDEGs was validated with 39 pairs HCC and paracarcinoma tissues by quantitative reverse transcription-PCR (qRT-PCR).

**Results:**

A total of 18 mitophagy genes that were upregulated and contributed to poor prognosis in HCC were identified. These genes could interact with each other. The correlation analysis showed that there was positively correlation among mitophagy genes. According to optimal *λ* value, 8 mitophagy gene signatures were involved in prognostic model. Based on median risk scores, HCC patients were divided into high-risk group and low-risk group. Compared with the low-risk group, the high-risk group has worse overall survival in TCGA cohort and ICGC cohort. The univariate and multivariate Cox regression analysis suggested that risk score was an independent prognostic factor of HCC patients. Time-dependent ROC curve was used to identify and validate good predicting performance of the prognostic model. Enrichment analysis showed that risk differentially expressed genes were enriched in various metabolism and cell division processes. The immune cell infiltration score and immune function were significantly different in two groups. qRT-PCR validation result showed that QSTM1, CSNK2B, PGAM5, and ATG5 were upregulated.

**Conclusion:**

Mitophagy genes could influence HCC progression through regulating the metabolism and immune functions and could be used to predict prognosis and considered as potential prognostic biomarker and precise therapeutic target of HCC.

## 1. Introduction

Hepatocellular carcinoma (HCC) is the sixth most common malignancy and the fourth most fatal tumor [1]. One reason for the high fatality rate lies in its unclear pathogenesis and the lack of early diagnostic and prognostic biomarkers as well as molecular therapeutic targets.

As we all know, mitochondria are the energy factories of cells and generate the most of the energy required by human. Mitochondria play a vital important role in apoptosis, necrosis, autophagy, stress regulation, lipid and carbohydrate production, Ca^2+^ storage, and innate immunity of cells [2]. Mitochondrial dynamic equilibrium, including mitochondria fission and fusion and mitophagy, was regulated by a series of complex mechanisms, thus maintaining the relative homeostasis of mitochondria to provide energy for cell function, cell repair, and regeneration under physiological conditions [3, 4]. As a highly conserved cellular process, mitophagy was mainly involved in degradation and eliminating aging or impaired mitochondria [5]. Mitophagy, as one of the important mechanisms for mitochondrial quality control, plays an indispensable role in regulating mitochondrial dynamics equilibrium and maintaining a normal physiological state [3].

Research suggested that mitophagy is a double-edged sword in tumor cells, and mitophagy usually could inhibit tumorigenesis by reducing dysfunctional mitochondrial accumulation, cellular oxidative stress, genomic instability, and inflammation [6]. Once carcinogenesis begins, mitophagy was highly activated to maintain the metabolic needs of tumor cells and promote tumor progression [6, 7]. These conclusions showed that mitophagy disequilibrium is critical for the occurrence and progression of tumor cells. At present, it has been reported that mitophagy genes were abnormally expressed and could modulate biological behaviors and drug susceptibility in HCC cells [8]. Autophagy mediated by mitophagy genes could prevent HCC oncogenesis by inhibiting the activation of inflammasome, which hinted that mitophagy could play important role in antitumor immunity of HCC [9]. Accordingly, we could conclude that mitophagy has an influence on the occurrence and development of HCC, but it is not yet known and remains to be explored that the specific role of mitophagy genes is whether promoting or suppressing tumor.

There is no systematically reported about the dysregulated profiles and prognostic characteristic of mitophagy genes in HCC. In this study, the expression and clinical information data of HCC in The Cancer Genome Atlas (TCGA) cohort and International Cancer Genome Consortium (ICGC) cohort were used to construct and externally validate prognostic model of mitophagy genes, detect its performance for predicting prognosis, illustrate its correlation with immune cell infiltration, and validate the mitophagy differentially expressed genes (MDEGs) with HCC tissues by quantitative reverse transcription-PCR (qRT-PCR). This study discloses the potential of mitophagy genes as prognostic biomarkers and precise therapeutic targets of HCC and provides important clues with mitochondrial pathogenesis in HCC.

## 2. Materials and Methods

### 2.1. Data Collection

The expression data of 365 HCC tissues was downloaded from TCGA database (https://cancergenome.nih.gov/), and the association clinical information was obtained from UCSC XENA (https://xenabrowser.net/). In addition, RNA sequencing data and clinical information of 231 HCC patients were gained from ICGC database (https://dcc.icgc.org/projects/LIRI-JP). The mitophagy gene set was downloaded from Molecular Signatures Database (MSigDB) [10].

### 2.2. Identification of MDEGs with Prognostic Values

Firstly, the MDEGs were identified in HCC and paracarcinoma tissue of TCGA cohort by “limma” R package (*P* < 0.05). Next, mitophagy genes with prognostic values were screened by the univariate Cox analysis of overall survival. Finally, the MDEGs with prognostic value were obtained by intersecting above two factors. The heatmap and prognostic forest map were visualized by “pheatmap” and “survival” R packages, respectively.

### 2.3. Interaction and Correlation Analysis among Mitophagy Genes

The protein-protein interaction network of MDEGs with prognostic value was constructed and visualized by STRING online database (https://string-db.org/). The association among mitophagy genes were analyzed and visualized in TCGA cohort by “igraph” and “reshape2” R package.

### 2.4. Construction and Validation of a Prognostic Mitophagy Gene Signature

The prognostic model of mitophagy genes in HCC was constructed by least absolute shrinkage and selection operator- (LASSO-) penalized Cox regression analysis [11]. LASSO algorithm was used to select and shrink variables. The independent variable in the regression was the standardized expression matrix of MDEGs with prognostic value, and the dependent variables were overall survival and survival status of HCC patients in the cohort. Penalty parameter (*λ*) was identified based on tenfold cross-validation following the minimum criteria. The risk score of patients was determined according to the standardized expression amount of each mitophagy gene and its corresponding regression coefficient and the specific formula is as follows:

(1) $$\begin{array}{c} \text{Risk score}=\overset{n}{\underset{i=1}{\sum }}\left(\text{Ex}{\text{p}}_{i}*\beta_{i}\right). \end{array}$$

*n* represents mitophagy genes number; *Exp*_*i*_ represents the expression level of each gene *i*; *β*_*i*_ was regression coefficient of gene *i* calculated by LASSO regression analysis. Patients were divided into high-risk group and low-risk group based on median risk score. The overall survival between two groups was performed by “surviminer” R package. Time-dependent receiver operator characteristic (ROC) curve was performed to evaluate the predicting performance of gene signatures by “survivalROC.” In addition, principal component analysis (PCA) and t-distributed stochastic neighbor embedding (t-SNE) was performed by “stats” and “Rtsne” R package. TCGA cohort was considered as train group and ICGC cohort was listed as test group, and the analysis methods of the two groups were completely consistent.

### 2.5. Enrichment Analysis and Immune Cell Infiltration between Different Risk Groups

The MDEGs between high-risk group and low-risk group were confirmed by “limma” R package (|log2FC| ≥ 1, *P* < 0.05). Gene Ontology (GO) and Kyoto Encyclopedia of Genes and Genomes (KEGG) analyses of MDEGs were carried out by “clusterProfiler” R package. Single-sample gene set enrichment analysis (ssGSEA) was used to assess 16 immune cell infiltration scores and 13 immune-related pathway activities between two groups.

### 2.6. qRT-PCR Validation In Vivo

39 pairs of HCC and paracarcinoma tissues were collected from The First Affiliated Hospital of China Medical University. This study was approved by the Ethics Committee of the First Affiliated Hospital of China Medical University. Every participant has signed informed consent before collecting the specimens. Total RNA was extracted, and quantitative reverse transcription-PCR experiments were performed using a real-time PCR 480 system and SuperReal PreMix Plus (SYBR Green, TIANGEN). All curves were a single peak. With *β*-actin as a normalization, mitophagy-related genes were detected in 39 HCC tissues (*n* = 39). The primer sequence is listed in Table S1.

### 2.7. Statistical Analysis

Student's *t*-test was used to identify MDEGs in HCC and paracarcinoma tissues. Chi-square test was utilized to compare clinicopathological characteristics between the high-risk group and low-risk group. The Mann-Whitney test calculated ssGSEA score of immune cell and immune-related pathway in two groups. Log-rank test compared overall survival of different groups. The univariate and multivariate Cox regression analysis was used to identify independent prognostic factor. 2^−*Δ*Ct^ represents relative expression level, and differential expression profiles were assessed by Student's *t*-test for normally distributed data, while a rank sum test was used for skewed distribution data. RStudio 3.6.1, SPSS (version 25.0), and GraphPad Prism V8.0 were used to perform data analysis and *P* < 0.05 is statistically significant.

## 3. Results

### 3.1. Identification of Prognostic Mitophagy DEGs in the TCGA Cohort

In this study, a total of 365 and 231 HCC patients were included from TCGA cohort and ICGC cohort, respectively. The clinicopathological characteristics of these patients are concluded in Table 1. Among the patients from TCGA, 46.6% were in stage I, 23.0% in stage II, 22.7% in stage III, and 1.1% in stage IV. Among TCGA patients, 15.1% of the patients had grade I, 47.9% had grade II, 32.3% had grade III, and 3.3% had grade IV. 15.6% of the ICGC patients were in stage I, and the percentages of patients in stage II, stage III, and stage IV were 45.5%, 30.7%, and 8.2%, respectively. However, the ICGC data did not provide grade information of the patients. The mean age of the patients was 61 and 69 years in TCGA and ICGC cohort, respectively.

**Figure 1:**
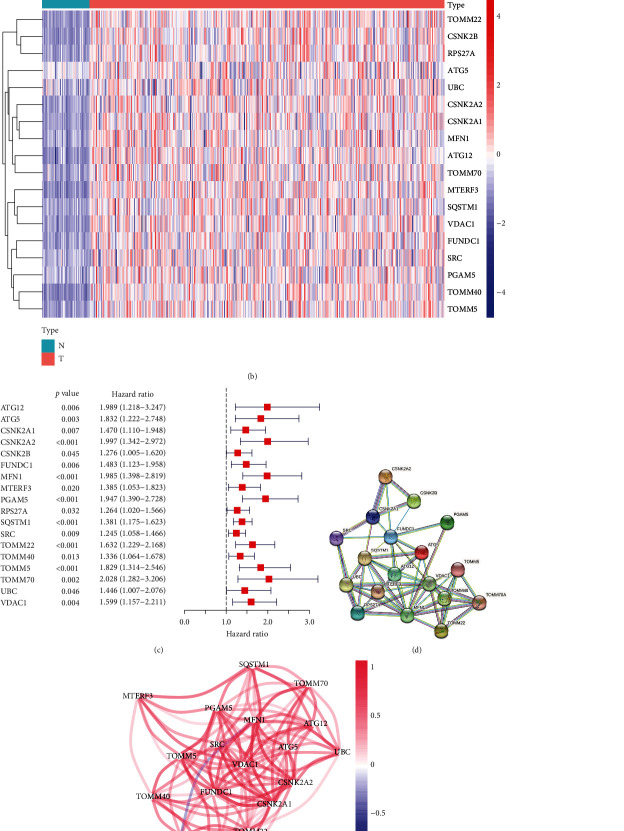
Identification of the prognostic MDEGs in TCGA cohort. (a) The intersect mitophagy genes between DEGs and prognostic genes. (b) The differentially expressed heatmap of prognostic mitophagy genes. (c) The univariate Cox regression analysis of prognostic MDEGs. (d) The protein-protein interaction network of prognostic MDEGs. (e) The correlation among prognostic MDEGs. HCC: hepatocellular carcinoma; MDEGs: mitophagy differentially expressed genes; TCGA: The Cancer Genome Atlas.

A total of 29 mitophagy genes were collected from MSigDB (Table S2), of which 25 genes was differentially expressed (Table S3) and 19 genes could become prognostic factor of HCC (Table S4). Further, the intersection of differential expression and prognosis-related genes was identified, and 18 mitophagy genes were upregulated and poor prognosis for HCC (Figures 1(a)–1(c)). These genes have multiple protein-protein interaction relationships (Figure 1(d)). Correlation analysis showed that these genes were positive with each other (Figure 1(e)).

### 3.2. Construction of a Prognostic Signature in the TCGA Cohort

**Figure 2:**
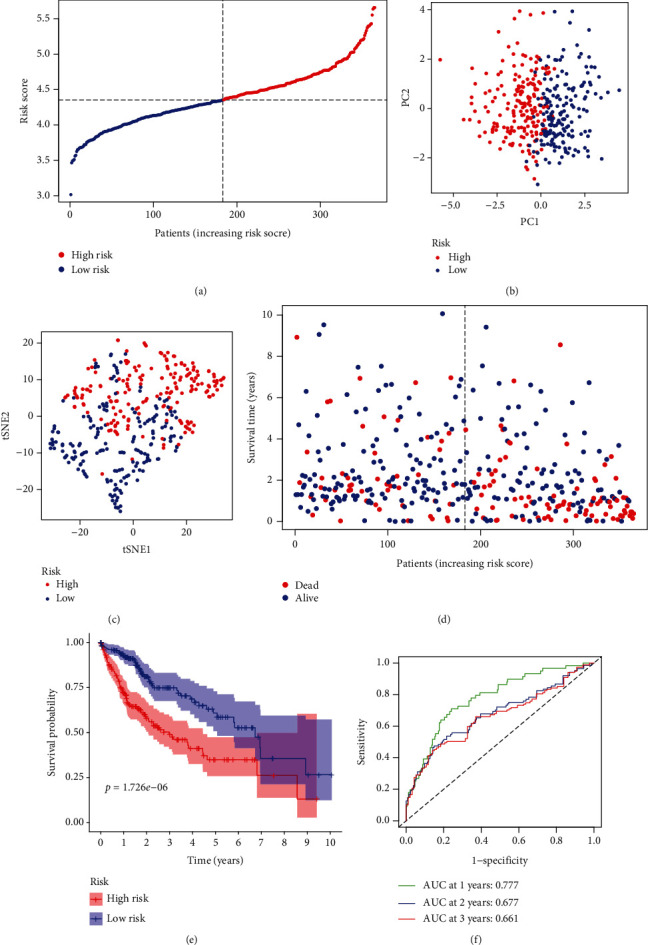
Prognostic characteristic of the 8-gene signature model in the TCGA cohort. (a) Distribution and median values of risk scores in the TCGA cohort. (b) PCA between the high-risk group and low-risk group in the TCGA cohort. (c) t-SNE analysis between the high-risk group and low-risk group in the TCGA cohort. (d) With the increasing of risk scores, the distribution of patient survival status in the TCGA cohort. (e) Overall survival of patients between the high-risk group and low-risk group in the TCGA cohort. (f) AUC of time-dependent ROC curves verified the prognostic performance of the risk score in the TCGA cohort. AUC: area under curve; PCA: principal component analysis; ROC: receiver operating characteristic curve; t-SNE, t-distributed stochastic neighbor embedding; TCGA: The Cancer Genome Atlas.

The expression profiles of 18 mitophagy genes were determined to construct a prognostic model by LASSO regression. Finally, based on the optimal *λ* value, 8 mitophagy genes were used to build a prognostic signature, including ATG5, CSNK2B, MFN1, PGAM5, SQSTM1, TOMM5, TOMM22, and TOMM70. According to median risk score, patients were divided into the high-risk group (*n* = 182) and low-risk group (*n* = 183) (Figure 2(a)). The high-risk group was closely related to pathology grade and was critically correlated with stage and depth of invasion in TCGA cohort (Table 2). PCA and t-SNE showed that the patients in two groups were distributed in two directions (Figures 2(b) and 2(c)). The mortality of patients the in high-risk group was higher than the low-risk group (Figure 2(d)). Meanwhile, the Kaplan-Meier curve suggested that the patients of the high-risk group have lower overall survival (Figure 2(e)). Time-dependent ROC curve was used to detect the predicting performance of prognostic model, and the result indicated that the area under the curve (AUC) reached 0.777 at 1 year, 0.677 at 2 years, and 0.661 at 3 years (Figure 2(f)).

### 3.3. Validation of a Prognostic Model in the ICGC Cohort

**Figure 3:**
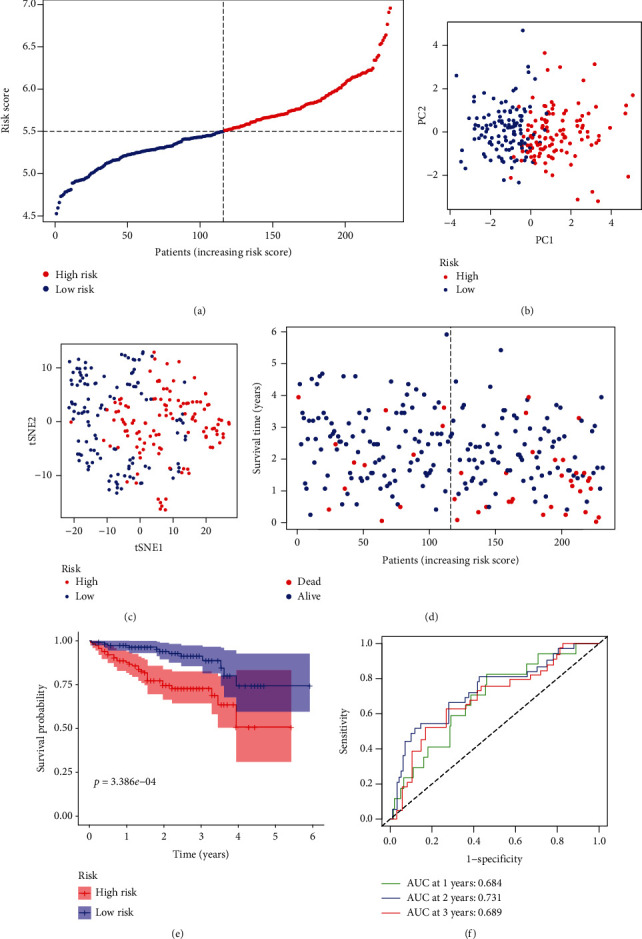
Validation prognostic characteristic of the 8-gene signature model in the ICGC cohort. (a) Distribution and median values of risk scores in the ICGC cohort. (b) PCA between the high-risk group and low-risk group in the ICGC cohort. (c) t-SNE analysis between the high-risk group and low-risk group in the ICGC cohort. (d) With the increasing of risk scores, the distribution of patient survival status in the ICGC cohort. (e) Overall survival of patients between the high-risk group and low-risk group in the ICGC cohort. (f) AUC of time-dependent ROC curves verified the prognostic performance of the risk score in the ICGC cohort. AUC: area under curve; PCA: principal component analysis; ROC: receiver operating characteristic curve; t-SNE: t-distributed stochastic neighbor embedding; ICGC: International Cancer Genome Consortium.

To test the stability of the model construction with TCGA cohort, ICGC cohort was performed completely consistent analysis to validate above results. The patients were also categorized into two groups based on the median risk score. The high-risk group of HCC patients was tightly associated with TNM stage in the ICGC cohort (Figure 3(a) and Table 2). Similarly, PCA and t-SNE suggested that two groups of patients were also distributed in different directions (Figures 3(b) and 3(c)). Survival analysis indicated that the prognosis of the high-risk group was worse than the low-risk group (Figures 3(d) and 3(e)). The AUC was 0.684 at 1 year, 0.731 at 2 years, and 0.689 at 3 years (Figure 3(f)).

### 3.4. Independent Prognostic Value of Mitophagy Gene Signatures

**Figure 4:**
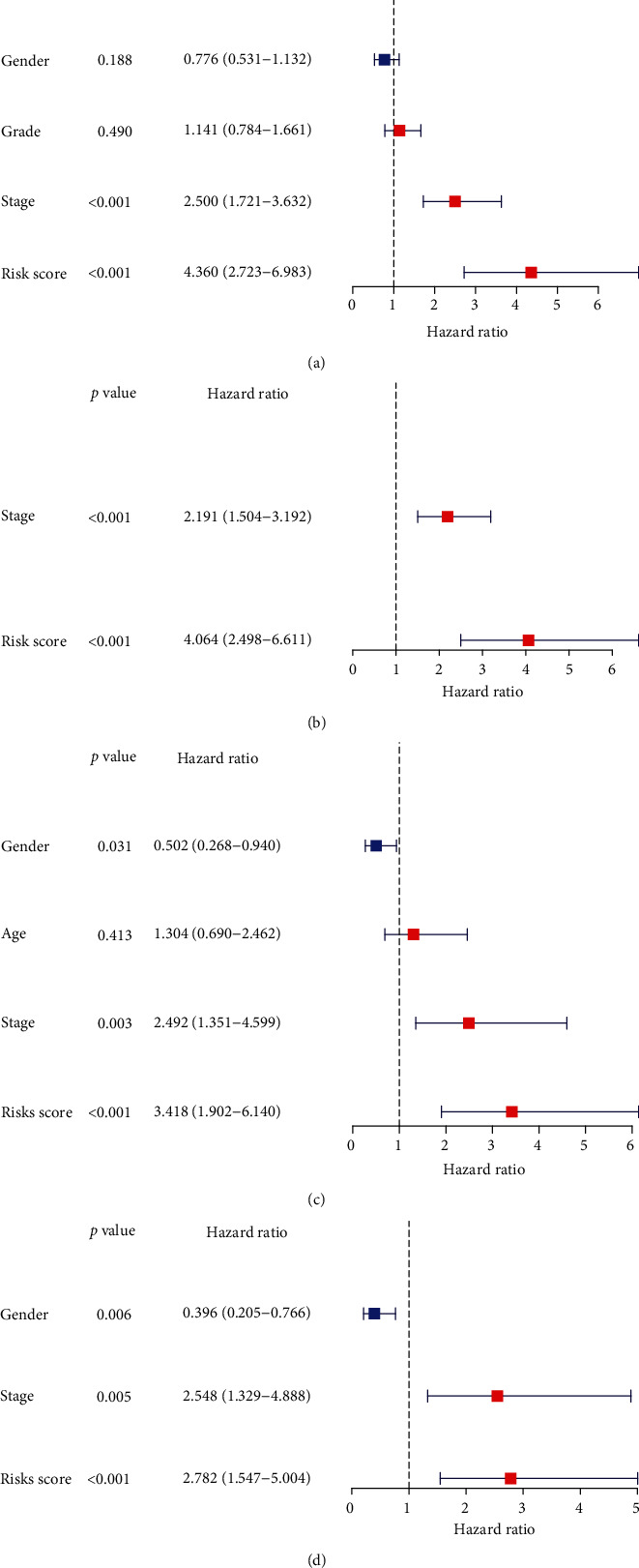
Univariate and multivariate Cox regression analyses for OS in the TCGA train group and ICGC test group. (a) Univariate Cox regression analyses in the TCGA train group. (b) Multivariate Cox regression analyses in the TCGA train group. (c) Univariate Cox regression analyses in the ICGC test group. (d) Multivariate Cox regression analyses in the ICGC test group. OS: overall survival; ICGC: International Cancer Genome Consortium; TCGA: The Cancer Genome Atlas.

To determine whether the risk score of mitophagy genes is an independent prognostic factor for HCC patients, the univariate and multivariate Cox regression analyses were performed in two cohorts, respectively. The univariate Cox analysis showed that stage (*P* < 0.001) and risk score (*P* < 0.001) were related to poor prognosis in TCGA cohort (Figure 4(a)). Further, the multivariate Cox analysis indicated that two parameters were independent prognostic factor (Figure 4(b)). Univariate analysis hinted that age (*P* = 0.031), stage (*P* = 0.003), and risk score (*P* < 0.001) were associated with poor overall survival (Figure 4(c)), and multivariate analysis disclosed that these parameters were also independent prognostic factors of HCC patients in ICGC (Figure 4(d)). The results of two cohorts were consistent showing that risk score was independent prognostic factor.

### 3.5. Function Analysis of MDEGs in Two Groups

**Figure 5:**
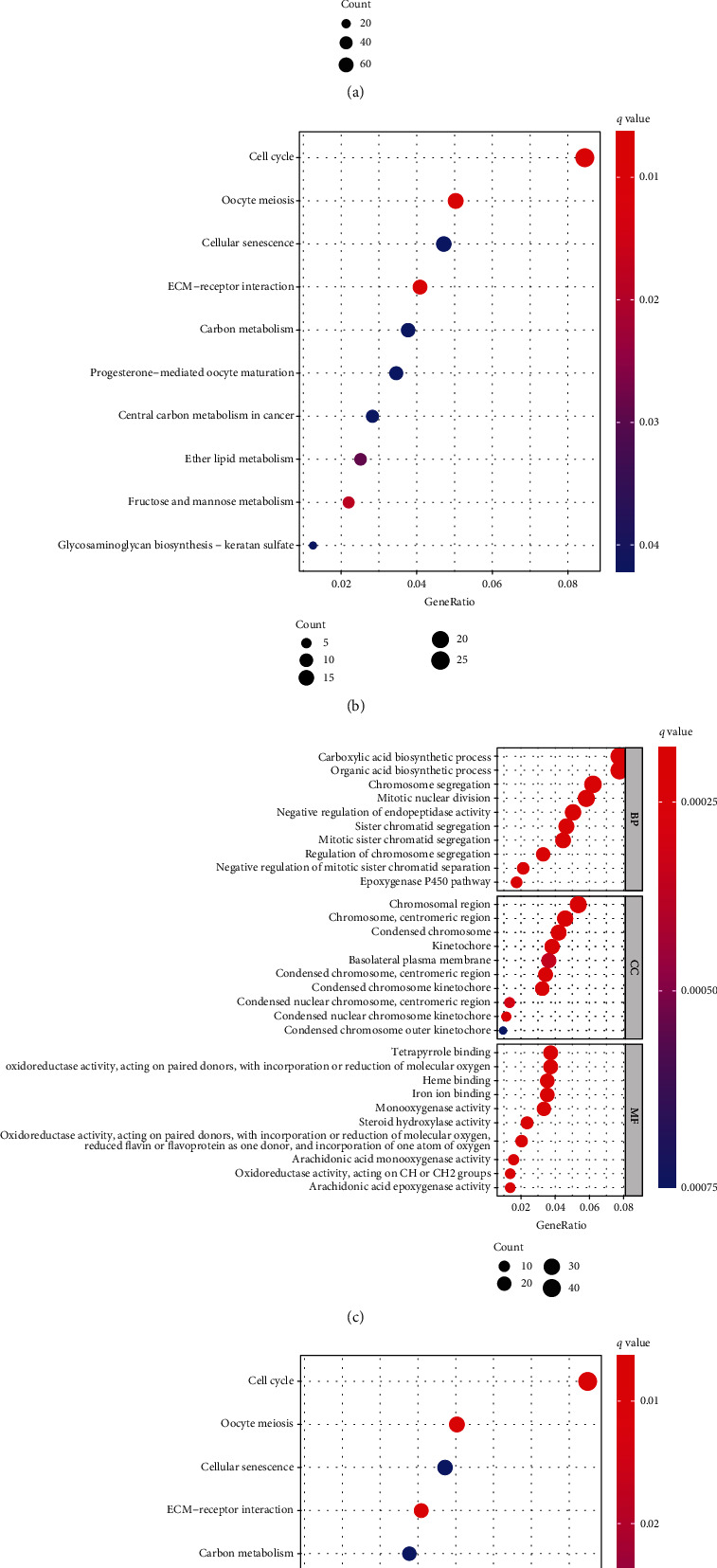
GO and KEGG analyses of differentially expressed genes in the high-risk group and low-risk group in the TCGA cohort (a and b) and ICGC cohort (c and d). (a) GO analysis in TCGA cohort. (b) KEGG analysis in TCGA cohort. (c) GO analysis in ICGC cohort. (d) KEGG analysis in ICGC cohort. GO: Gene Ontology; KEGG: Kyoto Encyclopedia of Genes and Genomes; ICGC: International Cancer Genome Consortium; TCGA: The Cancer Genome Atlas.

MDEGs were determined in two groups to perform GO and KEGG analyses to expound their potential function. The results in TCGA cohort showed that risk differentially expressed genes were mainly enriched in multiple metabolism and cell division processes (Figures 5(a) and 5(b)) and many pathways were validated in ICGC cohort, such as cell cycle, carbon metabolism, fructose, and mannose metabolism (Figures 5(c) and 5(d)).

**Figure 6:**
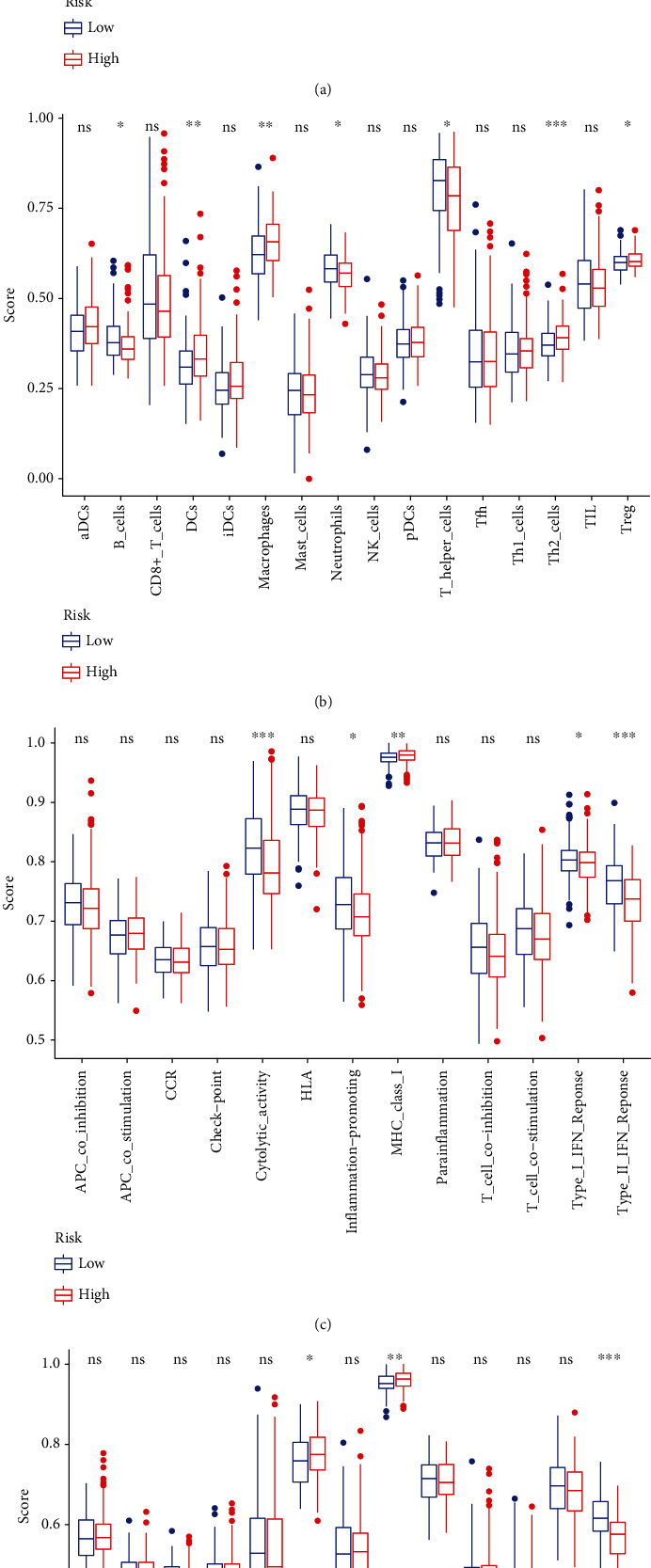
The scores of immune cells and immune-related function of differentially expressed genes in the high-risk group and low-risk group in the TCGA cohort (a and b) and ICGC cohort (c and d). (a) The scores of immune cells in TCGA cohort. (b) The scores of immune cells in ICGC cohort. (c) The immune-related function in TCGA cohort. (d) The immune-related function in ICGC cohort. ICGC: International Cancer Genome Consortium; TCGA: The Cancer Genome Atlas.

Mitophagy, as a powerful means modulating immune system, may limit the secretion of inflammatory cytokines and directly regulate mitochondrial antigen presentation and immune cell homeostasis [12, 13]. Therefore, we explored the association of risk score with immune status, and the results demonstrated that the immune scores of B cell, DCs, macrophages, neutrophils, and T helper cell were significantly different in the high-risk group and low-risk group of TCGA cohort and ICGC cohort (Figures 6(a) and 6(b)). Compared with the low-risk group, the proportion of B cell, DCs, neutrophils, and T helper cell was lower, while macrophages were higher in the high-risk group. The change of pathway activity was statistically significant (Figures 6(c) and 6(d)), and the proportion of MHC_class_I was decreased, while Type_II_IFN_Reponse was increased in the high-risk group.

### 3.6. qRT-PCR Validation of 8-Prognostic Signature in HCC

**Figure 7:**
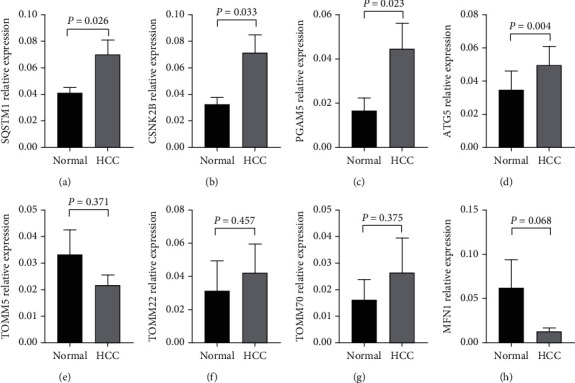
qRT-PCR validation in hepatocellular carcinoma tissues (*n* = 39). (a) SQSTM1. (b) CSNK2B. (c) PGAM5. (d) ATG5. (e) TOMM5. (f) TOMM22. (g) TOMM70. (h) MFN1. Measurement data were expressed as mean ± SEM. qRT-PCR: quantitative real-time polymerase chain reaction. SEM: standard error of median.

39 pairs HCC and paracarcinoma tissues were used to validate 8 mitophagy genes in prognostic model, including SQSTM1, CSNK2B, PGAM5, ATG5, TOMM5, TOMM22, TOMM70, and MFN1. qRT-PCR results showed that SQSTM1 (*P* = 0.026), CSNK2B (*P* = 0.033), PGAM5 (*P* = 0.023), and ATG5 (*P* = 0.004) were upregulated, while TOMM5, TOMM22, TOMM70, and MFN1 have no significance (*P* > 0.05, Figure 7).

## 4. Discussion

This study analyzed the expression profile and the association with overall survival of 29 mitophagy genes in HCC. Finally, 8 mitophagy genes were used to construct prognostic signature and perform external and experimental validation. Enrichment analysis showed that the signature was mainly enriched in metabolism and cell division processes and was tightly related to immune cell infiltration.

It is reported that mitophagy genes have an influence on HCC progression and prognosis [14, 15]. This study initially constructed and externally validated the prognostic model consisting of mitophagy gene signatures with TCGA cohort and ICGC cohort. Finally, 8 mitophagy genes that were upregulated and prognostic related were included in the model, including ATG5, CSNK2B, MFN1, PGAM5, SQSTM1, TOMM5, TOMM22, and TOMM70. qRT-PCR validation of 39 pairs HCC and paracarcinoma tissues proved that SQSTM1 (*P* = 0.026), CSNK2B (*P* = 0.033), PGAM5 (*P* = 0.023), and ATG5 (*P* = 0.004) were upregulated. The research suggested that high expression of ATG5 could promote mitophagy to decrease the sensitivity to sorafenib for HCC [16]. CSNK2B was downregulated by tumor necrosis factor-*α*-inducible protein 1 to inhibit the activation of nuclear factor-*κ*B, thus modulating proliferation, migration, and angiogenesis of HCC [17]. PGAM5 dissociated from BCL-xL could control mitochondrial fission, mitophagy, and tumor cell apoptosis through FUNDC1 pathway [18]. SQSTM1 was upregulated and associated with poor prognosis [19] as well as regulated the occurrence and development of tumor through various mechanisms [20, 21]. TOMM22 phosphorylated by CSNK2 could regulate mitochondrial homeostasis by modulating mitophagy [22]. The interaction role of MFN1 and PINK induced dysregulation of mitophagy process which contributed to glucose-induced pathological epithelial-stromal transformation in tumor cells [23]. TOMM machinery is a molecular switch for the mitophagy process dependent on PINK1 and PARK2/PARKIN [24]. It has been seen that 8 gene signatures involved in the model were closely related to mitophagy process. This study built prognostic model of MDEGs for the first time. According to median risk score, patients were divided into the high-risk group and low-risk group. Both TCGA cohort and ICGC cohort indicated that the patients of the high-risk group have lower overall survival than the low-risk group, time-dependent ROC curve proved that the predicting performance of the model was good, and risk score could be considered as independent poor prognostic factor.

Enrichment analysis was performed based on the difference in two risk groups. We found that the risk differential genes were mainly enriched in various metabolism and cell mitosis processes. It has been reported that mitophagy disequilibrium could result in accumulating abnormal mitochondria, decreasing oxidative phosphorylation, and enhancing reactive oxygen species production and glycolysis, thus promoting the Warburg effect to cause tumorigenesis [25]. The stasis of mitosis process may lead to enhance mitophagy and reduce mitochondrial mass accompanied by ATP reduction, which could have an effect on tumor progression [26, 27]. This study also proved that risk differential genes could play role in cell metabolism and mitosis, which hinted that mitophagy genes modulate tumor progression through regulating these metabolism functions.

Existing research proves that mitophagy triggers an adaptive immune response during tumorigenesis [28, 29]. Therefore, we explored the association of risk score with immune status. Compared with the low-risk group, the proportion of B cell, DCs, neutrophils, and T helper cell was lower, while macrophages were higher in the high-risk group. The proportion of MHC_class_I was decreased, while Type_II_IFN_Reponse was increased for immune function in the high-risk group. The higher the abundance of B cell and T cell infiltration, the better the clinical outcome of HCC [30, 31]. DG infiltration was positive with disease-free survival [32]. M2 macrophages could promote tumor invasion through epithelial mesenchymal transformation induced by CCL22 [33]. MHC_class_I molecular could express in HCC and regulate biological behaviors of tumor cells [34]. We can conclude that these immune cells and molecular have an influence on HCC prognosis. This study found that these immune cells were significantly different in the high-risk group and low-risk group, which reminded that these gene signatures influence progression and prognosis of HCC through immune cell mass and mitophagy.

In conclusion, the expression and clinical data of TCGA cohort and ICGC cohort were used to construct and externally validate the prognostic model, detect its predicting performance, illustrate their potential pathological mechanisms, disclose the potency of mitophagy genes as prognostic biomarkers and precise therapeutic targets, and lay a foundation and provide detailed data analysis for subsequent mechanism research.

## Figures and Tables

**Table 1:** Clinical characteristics of the HCC patients in TCGA and ICGC cohort.

Variables	TCGA cohort	ICGC cohort
Cases	365	231
Age (median, range)	61 (16-90)	69 (31-89)
Gender (%)		
Female	119 (32.6%)	61 (26.4)
Male	246 (67.4%)	170 (73.6)
Grade (%)		
G1	55 (15.1)	NA
G2	175 (47.9)	NA
G3	118 (32.3)	NA
G4	12 (3.3)	NA
Unknown	5 (1.4)	NA
Stage (%)		
I	170 (46.6)	36 (15.6)
II	84 (23.0)	105 (45.5)
III	83 (22.7)	71 (30.7)
IV	4 (1.1)	19 (8.2)
Unknown	24 (6.6)	0 (0.0)
Survival status		
OS days (median)	596	780
Alive	235 (64.4)	189 (81.8)
Dead	130 (35.6)	42 (18.2)

HCC: hepatocellular carcinoma; ICGC: International Cancer Genome Consortium; TCGA: The Cancer Genome Atlas.

**Table 2:** Baseline characteristics of HCC patients in different risk groups.

		TCGA cohort				ICGC cohort		
Variables	Cases	High risk *n* (%)	Low risk *n* (%)	*P* value	Cases	High risk *n* (%)	Low risk *n* (%)	*P* value
Gender	365				231			
Male	246	128 (52.0)	118 (48.0)		170	88 (51.8)	82 (48.2)	
Female	119	54 (45.4)	65 (54.6)	0.233	61	27 (44.3)	34 (55.7)	0.315
Age (years)	365				231			
>65	138	68 (49.3)	70 (50.7)		142	72 (50.7)	70 (49.3)	
≤65	227	114 (50.2)	113 (49.8)	0.816	89	43 (48.3)	46 (51.7)	0.724
Grade	360				—			
G1+G2	230	96 (41.7)	134 (58.3)		—	—	—	
G3+G4	130	84 (64.6)	46 (35.4)	<0.001	—	—	—	—
Invasive extent	362				—			
T1-2	271	129 (47.6)	142 (52.4)		—	—	—	
T3-4	91	53 (58.2)	38 (41.8)	0.079	—	—	—	—
TNM stage	341				231			
I+II	254	119 (46.9)	135 (53.1)		141	60 (42.6)	81 (57.4)	
III+IV	87	50 (57.5)	37 (42.5)	0.087	90	55 (61.1)	35 (38.9)	0.006

HCC: hepatocellular carcinoma; ICGC: International Cancer Genome Consortium; TCGA: The Cancer Genome Atlas.

## Data Availability

The data that support the results of this manuscript are available from the corresponding author upon reasonable request.

## Abbreviations

HCC: Hepatocellular carcinoma
LASSO: Least absolute shrinkage and selection operator
TCGA: The Cancer Genome Atlas
ICGC: International Cancer Genome Consortium
MDEGs: Mitophagy differentially expressed genes
ROC: Receiver operating characteristic
GO: Gene Ontology
KEGG: Kyoto Encyclopedia of Genes and Genomes
ssGSEA: Single-sample gene set enrichment analysis
PCA: Principal component analysis
t-SNE: t-distributed stochastic neighbor embedding
AUC: Area under the curve
qRT-PCR: Quantitative real-time polymerase chain reaction.

## Supplementary Materials

Supplementary Materials Table S1: the primer sequence used for qRT-PCR. Table S2: the description of mitophagy genes. Table S3: the differentially expressed mitophagy genes in HCC with TCGA data. Table S4: the prognostic value of mitophagy genes in HCC with TCGA data.

## References

[1] Villanueva A. (2019). Hepatocellular carcinoma. *The New England Journal of Medicine*.

[2] Galluzzi L., Kepp O., Trojel-Hansen C., Kroemer G. (2012). Mitochondrial control of cellular life, stress, and death. *Circulation Research*.

[3] Wu N. N., Zhang Y., Ren J. (2019). Mitophagy, mitochondrial dynamics, and homeostasis in cardiovascular aging. *Oxidative Medicine and Cellular Longevity*.

[4] Bhargava P., Schnellmann R. G. (2017). Mitochondrial energetics in the kidney. *Nature Reviews Nephrology*.

[5] Zong W. X., Rabinowitz J. D., White E. (2016). Mitochondria and cancer. *Molecular Cell*.

[6] Ma X., McKeen T., Zhang J., Ding W. X. (2020). Role and mechanisms of mitophagy in liver diseases. *Cells*.

[7] Panigrahi D. P., Praharaj P. P., Bhol C. S. (2020). The emerging, multifaceted role of mitophagy in cancer and cancer therapeutics. *Seminars in Cancer Biology*.

[8] Zheng Y., Huang C., Lu L. (2021). STOML2 potentiates metastasis of hepatocellular carcinoma by promoting PINK1-mediated mitophagy and regulates sensitivity to lenvatinib. *Journal of Hematology & Oncology*.

[9] Li W., Li Y., Siraj S. (2019). FUN14 domain-containing 1-mediated mitophagy suppresses hepatocarcinogenesis by inhibition of inflammasome activation in mice. *Hepatology (Baltimore, Md)*.

[10] Subramanian A., Tamayo P., Mootha V. K. (2005). Gene set enrichment analysis: a knowledge-based approach for interpreting genome-wide expression profiles. *Proceedings of the National Academy of Sciences of the United States of America*.

[11] Simon N., Friedman J., Hastie T., Tibshirani R. (2011). Regularization paths for Cox's proportional hazards model via coordinate descent. *Journal of Statistical Software*.

[12] Xu Y., Shen J., Ran Z. (2020). Emerging views of mitophagy in immunity and autoimmune diseases. *Autophagy*.

[13] Song Y., Zhou Y., Zhou X. (2020). The role of mitophagy in innate immune responses triggered by mitochondrial stress. *Cell Communication and Signaling : CCS*.

[14] Zheng J., Chen L., Lu T. (2020). MSCs ameliorate hepatocellular apoptosis mediated by PINK1-dependent mitophagy in liver ischemia/reperfusion injury through AMPK*α* activation. *Cell Death & Disease*.

[15] Chen Y., Chen H. N., Wang K. (2019). Ketoconazole exacerbates mitophagy to induce apoptosis by downregulating cyclooxygenase-2 in hepatocellular carcinoma. *Journal of Hepatology*.

[16] Zhang K., Chen J., Zhou H. (2018). PU.1/microRNA-142-3p targets ATG5/ATG16L1 to inactivate autophagy and sensitize hepatocellular carcinoma cells to sorafenib. *Cell Death & Disease*.

[17] Xiao Y., Huang S., Qiu F. (2020). Tumor necrosis factor *α*-induced protein 1 as a novel tumor suppressor through selective downregulation of CSNK2B blocks nuclear factor-*κ*B activation in hepatocellular carcinoma. *eBioMedicine*.

[18] Ma K., Zhang Z., Chang R. (2020). Dynamic PGAM5 multimers dephosphorylate BCL-xL or FUNDC1 to regulate mitochondrial and cellular fate. *Cell Death and Differentiation*.

[19] Feng L., Chen M., Li Y. (2021). Sirt1 deacetylates and stabilizes p62 to promote hepato-carcinogenesis. *Cell Death & Disease*.

[20] Zhang H., Zhang Y., Zhu X. (2019). DEAD box protein 5 inhibits liver tumorigenesis by stimulating autophagy via interaction with p62/SQSTM1. *Hepatology (Baltimore, Md)*.

[21] Drake L. E., Springer M. Z., Poole L. P., Kim C. J., Macleod K. F. (2017). Expanding perspectives on the significance of mitophagy in cancer. *Seminars in Cancer Biology*.

[22] Kravic B., Harbauer A. B., Romanello V. (2018). In mammalian skeletal muscle, phosphorylation of TOMM22 by protein kinase CSNK2/CK2 controls mitophagy. *Autophagy*.

[23] Liu X., Feng C., Wei G. (2020). Mitofusin1 is a major mediator in glucose-induced epithelial-to-mesenchymal transition in lung adenocarcinoma cells. *OncoTargets and Therapy*.

[24] Bertolin G., Ferrando-Miguel R., Jacoupy M. (2013). The TOMM machinery is a molecular switch in PINK1 and PARK2/PARKIN-dependent mitochondrial clearance. *Autophagy*.

[25] Ferro F., Servais S., Besson P., Roger S., Dumas J. F., Brisson L. (2020). Autophagy and mitophagy in cancer metabolic remodelling. *Seminars in Cell & Developmental Biology*.

[26] Peña-Blanco A., Haschka M. D., Jenner A. (2020). Drp1 modulates mitochondrial stress responses to mitotic arrest. *Cell Death and Differentiation*.

[27] Doménech E., Maestre C., Esteban-Martínez L. (2015). AMPK and PFKFB3 mediate glycolysis and survival in response to mitophagy during mitotic arrest. *Nature Cell Biology*.

[28] Ziegler P. K., Bollrath J., Pallangyo C. K. (2018). Mitophagy in intestinal epithelial cells triggers adaptive immunity during tumorigenesis. *Cell*.

[29] O'Sullivan T. E., Johnson L. R., Kang H. H., Sun J. C. (2015). BNIP3- and BNIP3L-mediated mitophagy promotes the generation of natural killer cell memory. *Immunity*.

[30] Zhang Z., Ma L., Goswami S. (2019). Landscape of infiltrating B cells and their clinical significance in human hepatocellular carcinoma. *Oncoimmunology*.

[31] Chew V., Chen J., Lee D. (2012). Chemokine-driven lymphocyte infiltration: an early intratumoural event determining long-term survival in resectable hepatocellular carcinoma. *Gut*.

[32] Cai X. Y., Qiu S. J., Wu Z. Q. (2005). Relationship between dendritic cells and memory T lymphocytes in tumor site and prognosis of hepatocellular carcinoma. *Zhonghua Yi Xue Za Zhi*.

[33] Yeung O. W., Lo C. M., Ling C. C. (2015). Alternatively activated (M2) macrophages promote tumour growth and invasiveness in hepatocellular carcinoma. *Journal of Hepatology*.

[34] Deng X. L., Chen W., Cai M. Y., Wei D. P. (2003). Expression of class I MHC molecule, HSP70 and TAP in human hepatocellular carcinoma. *World Journal of Gastroenterology*.

