# Supplementary material for: Predicting Prognosis of Hepatocellular Carcinoma Patients Based on the Expression Signatures of Mitophagy Genes
Source: Dis Markers. 2022 Sep 16;2022:4835826. doi: 10.1155/2022/4835826 (PMC9507775; doi:10.1155/2022/4835826)
Supplement: Supplementary Materials — Table S1: the primer sequence used for qRT-PCR. Table S2: the description of mitophagy genes. Table S3: the differentially expressed mitophagy genes in HCC with TCGA data. Table S4: the prognostic value of mitophagy genes in HCC with TCGA data. [file 4835826.f1.docx]

**Supplementary materials**

**Predicting prognosis of** **hepatocellular carcinoma patients based on the expression signatures of mitophagy genes**

Yan-ke Li^1, 3^, Li-rong Yan^1^, Li-yue Jiang^1,4^, Qian Xu^1^, Ben-gang Wang^1, 2,^ *

^1^Institute of General Surgery, the First Affiliated Hospital of China Medical University, Shenyang 110001, China

^2^Department of Hepatobiliary Surgery, Institute of General Surgery, the First Hospital of China Medical University, Shenyang 110001, China

^3^Department of Anorectal Surgery, Institute of General Surgery, the First Hospital of China Medical University, Shenyang 110001, China

^4^ Tangdu Hospital of the Fourth Military Medical University, Xi-an 711032, China

***Corresponding author:** Dr. Ben-gang Wang, Department of Hepatobiliary Surgery, Institute of General Surgery, the First Hospital of China Medical University, North Nanjing Street 155#, Heping District, Shenyang110001, China. Telephone：+86-024-83283308; fax: +86-024-83282383. Email：[bgwang@cmu.edu.cn](mailto:bgwang@cmu.edu.cn)

| **Table S1. The primer sequence used for qRT-PCR** | |
| --- | --- |
| **Primer name** | **Sequence** |
| MFN1 |  |
| F | CGGAACTTGATCGAATAGCC |
| R | AGAGCTCTTCCCACTGCTTG |
| SQSTM1 |  |
| F | CTGCCTTCTTCCAGGATCAG |
| R | GTGAAAGCCATTAGGCAAGC |
| PGAM5 |  |
| F | GCCGGAAGCTGTGCAGTATT |
| R | GGTGGGTGATGCTGCCATTA |
| TOMM70 |  |
| F | CAATGCCAATGCAGCCAAAC |
| R | AGTGGACAGCAAAGGCTGCT |
| ATG5 |  |
| F | GCAACTCTGGATGGGATTGC |
| R | CAACTGTCCATCTGCAGCCA |
| CSNK2B |  |
| F | GCCCGCTACATCCTTACCAA |
| R | CTGGGATGTCTGAAAGGCCAAT |
| TOMM22 |  |
| F | ACGACGATGAGGAGCTAGATG |
| R | GGAAGTGGTCCCAATCCACA |
| TOMM5 |  |
| F | CTCCTGCGAGTCACTCCATT |
| R | GCCTATTCACTTGCAGAGAGG |
| β-actin |  |
| F | ATGTGGCCGAGGACTTTGATT |
| R | AGTGGGGTGGCTTTTAGGATG |
| qRT-PCR, quantitative real-time polymerase chain reaction. | |

| **Table S2. The description of mitophagy genes** | |
| --- | --- |
| **Mitophagy genes** | **Official full name** |
| ATG12 | autophagy related 12 |
| ATG5 | autophagy related 5 |
| CSNK2A1 | casein kinase 2 alpha 1 |
| CSNK2A2 | casein kinase 2 alpha 2 |
| CSNK2B | casein kinase 2 beta |
| FUNDC1 | FUN14 domain containing 1 |
| MAP1LC3A | microtubule associated protein 1 light chain 3 alpha |
| MAP1LC3B | microtubule associated protein 1 light chain 3 beta |
| MFN1 | mitofusin 1 |
| MFN2 | mitofusin 2 |
| MTERF3 | mitochondrial transcription termination factor 3 |
| PGAM5 | PGAM family member 5, mitochondrial serine/threonine protein phosphatase |
| PINK1 | PTEN induced kinase 1 |
| PRKN | parkin RBR E3 ubiquitin protein ligase |
| RPS27A | ribosomal protein S27a |
| SQSTM1 | sequestosome 1 |
| SRC | SRC proto-oncogene, non-receptor tyrosine kinase |
| TOMM20 | translocase of outer mitochondrial membrane 20 |
| TOMM22 | translocase of outer mitochondrial membrane 22 |
| TOMM40 | translocase of outer mitochondrial membrane 40 |
| TOMM5 | translocase of outer mitochondrial membrane 5 |
| TOMM6 | translocase of outer mitochondrial membrane 6 |
| TOMM7 | translocase of outer mitochondrial membrane 7 |
| TOMM70 | translocase of outer mitochondrial membrane 70 |
| UBA52 | ubiquitin A-52 residue ribosomal protein fusion product 1 |
| UBB | ubiquitin B |
| UBC | ubiquitin C |
| ULK1 | unc-51 like autophagy activating kinase 1 |
| VDAC1 | voltage dependent anion channel 1 |

| **Table S3. The differentially expressed mitophagy genes in HCC with TCGA data** | | | | | |
| --- | --- | --- | --- | --- | --- |
| **Gene** | **conMean** | **treatMean** | **LogFC** | ***P* value** | **FDR** |
| ATG12 | 2.154 | 3.804 | 0.821 | <0.001 | <0.001 |
| ATG5 | 4.758 | 5.667 | 0.252 | 0.002 | 0.002 |
| CSNK2A1 | 4.610 | 9.429 | 1.032 | <0.001 | <0.001 |
| CSNK2A2 | 3.766 | 6.376 | 0.760 | <0.001 | <0.001 |
| CSNK2B | 10.201 | 23.500 | 1.204 | <0.001 | <0.001 |
| FUNDC1 | 2.750 | 5.662 | 1.042 | <0.001 | <0.001 |
| MAP1LC3B | 6.993 | 8.887 | 0.346 | 0.013 | 0.014 |
| MFN1 | 1.957 | 3.592 | 0.876 | <0.001 | <0.001 |
| MTERF3 | 4.171 | 10.344 | 1.310 | <0.001 | <0.001 |
| PGAM5 | 6.968 | 10.905 | 0.646 | <0.001 | <0.001 |
| PINK1 | 9.085 | 6.857 | -0.406 | <0.001 | <0.001 |
| PRKN | 0.433 | 0.812 | 0.907 | 0.034 | 0.036 |
| RPS27A | 57.784 | 124.185 | 1.104 | <0.001 | <0.001 |
| SQSTM1 | 29.987 | 99.918 | 1.736 | <0.001 | <0.001 |
| SRC | 1.476 | 5.369 | 1.862 | <0.001 | <0.001 |
| TOMM20 | 24.224 | 59.980 | 1.308 | <0.001 | <0.001 |
| TOMM22 | 17.392 | 31.141 | 0.840 | <0.001 | <0.001 |
| TOMM40 | 6.780 | 19.510 | 1.525 | <0.001 | <0.001 |
| TOMM5 | 2.317 | 4.578 | 0.983 | <0.001 | <0.001 |
| TOMM7 | 36.743 | 62.782 | 0.773 | <0.001 | <0.001 |
| TOMM70 | 12.459 | 18.101 | 0.539 | <0.001 | <0.001 |
| UBA52 | 51.871 | 102.565 | 0.984 | <0.001 | <0.001 |
| UBC | 86.460 | 118.379 | 0.453 | <0.001 | <0.001 |
| ULK1 | 4.469 | 8.455 | 0.920 | <0.001 | <0.001 |
| VDAC1 | 41.603 | 76.237 | 0.874 | <0.001 | <0.001 |
| FDR, false discovery rate; FC, fold change; HCC, hepatocellular carcinoma; TCGA, The Cancer Genome Atlas. | | | | | |

| **Table S4. The prognostic value of mitophagy genes in HCC with TCGA data** | | | | |
| --- | --- | --- | --- | --- |
| **Gene** | **HR** | **HR.95 L** | **HR.95 H** | ***P* value** |
| ATG12 | 1.989 | 1.218 | 3.247 | 0.006 |
| ATG5 | 1.832 | 1.222 | 2.748 | 0.003 |
| CSNK2A1 | 1.470 | 1.110 | 1.948 | 0.007 |
| CSNK2A2 | 1.997 | 1.342 | 2.972 | 0.001 |
| CSNK2B | 1.276 | 1.005 | 1.620 | 0.045 |
| FUNDC1 | 1.483 | 1.123 | 1.958 | 0.006 |
| MFN1 | 1.985 | 1.398 | 2.819 | <0.001 |
| MTERF3 | 1.385 | 1.053 | 1.823 | 0.020 |
| PGAM5 | 1.947 | 1.390 | 2.728 | <0.001 |
| RPS27A | 1.264 | 1.020 | 1.566 | 0.032 |
| SQSTM1 | 1.381 | 1.175 | 1.623 | <0.001 |
| SRC | 1.245 | 1.058 | 1.466 | 0.009 |
| TOMM22 | 1.632 | 1.229 | 2.168 | 0.001 |
| TOMM40 | 1.336 | 1.064 | 1.678 | 0.013 |
| TOMM5 | 1.829 | 1.314 | 2.546 | <0.001 |
| TOMM70 | 2.028 | 1.282 | 3.206 | 0.002 |
| UBB | 0.663 | 0.477 | 0.922 | 0.014 |
| UBC | 1.446 | 1.007 | 2.076 | 0.046 |
| VDAC1 | 1.599 | 1.157 | 2.211 | 0.004 |
| HCC, hepatocellular carcinoma; HR, hazard ratio; H, high; L, low; TCGA, The Cancer Genome Atlas | | | | |
